# NanoModeler CG: A Tool for Modeling and Engineering Functional Nanoparticles at a Coarse-Grained Resolution

**DOI:** 10.1021/acs.jctc.2c01029

**Published:** 2023-02-16

**Authors:** Sebastian Franco-Ulloa, Laura Riccardi, Federico Rimembrana, Edwin Grottin, Mattia Pini, Marco De Vivo

**Affiliations:** † Molecular Modeling and Drug Discovery Lab, Istituto Italiano di Tecnologia, via Morego 30, Genova 16163, Italy; ‡ Expert Analytics, Møllergata 8, Oslo 0179, Norway

## Abstract

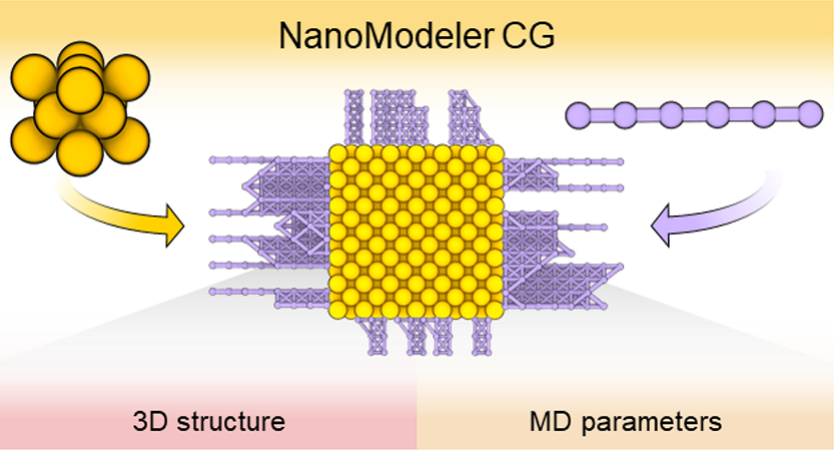

Functionalized metal nanoparticles (NPs) are macromolecular assemblies with a tunable physicochemical profile that makes them interesting for biotechnology, materials science, and energy conversion. In this regard, molecular simulations offer a way to scrutinize the structural and dynamical features of monolayer-protected NPs and their interactions with relevant matrices. Previously, we developed NanoModeler, a webserver that automates the preparation of functionalized gold NPs for atomistic molecular dynamics (MD) simulations. Here, we present NanoModeler CG (www.nanomodeler.it), a new release of NanoModeler that now also allows the building and parametrizing of monolayer-protected metal NPs at a coarse-grained (CG) resolution. This new version extends our original methodology to NPs of eight different core shapes, conformed by up to 800,000 beads and coated by eight different monolayer morphologies. The resulting topologies are compatible with the Martini force field but are easily extendable to any other set of parameters parsed by the user. Finally, we demonstrate NanoModeler CG’s capabilities by reproducing experimental structural features of alkylthiolated NPs and rationalizing the brush-to-mushroom phase transition of PEGylated anionic NPs. By automating the construction and parametrization of functionalized NPs, the NanoModeler series offers a standardized way to computationally model monolayer-protected nanosized systems.

## Introduction

Metal nanoparticles (NPs) are attracting interest because rapid advances in synthetic chemistry offer greater control over their structural and chemical features.^1^ Metal NPs have electronic and optical properties that are specific to their size, being absent in bulks of the same materials.^2^ Furthermore, their high area-to-volume ratios and ease of surface functionalization make metal NPs appealing candidates for many applications.^3^ Tailored metal NPs are now used in materials sciences,^4^ catalysis,^5−7^ drug delivery,^8−10^ and bioimaging,^11−13^ to name a few.

Metal NPs comprise an inner metallic core, to which a functionalizing monolayer is attached. The protecting monolayer is a supramolecular body comprising a collection of molecules, known as ligands. These ligands are typically bound to the core by thiols and amines (for noble metals), phosphine oxides and phosphonyls (for semiconducting quantum dots), or carboxyls and hydroxyls (for transition metal oxides).^14,15^ By modifying the surface chemistry of the pristine cores, the functionalizing ligands confer the NPs a characteristic charge distribution and hydrophobicity.^16^ These ultimately dictate the NP’s solubility, chemical stability, and interaction patterns with external entities.^17^ In other words, the protecting monolayer governs the physicochemical properties of metal NPs, thus influencing their effectiveness and transferability to applications in biomedicine, materials science, and energy conversion/storage.^18,19^

The knowledge-based design of functionalized metal NPs for custom applications requires an in-depth understanding of their structural and dynamical characteristics.^20−22^ Computational methods, in particular molecular dynamics (MD) simulations, are a versatile approach to this problem. MD simulations are especially useful as they allow researchers to study a controlled set of particles at a molecular scale for time intervals in the order of microseconds.^22−26^ MD simulations have been used to dissect the membrane translocation mechanism of mixed monolayer gold NPs,^27−30^ identify diverse binding modes of analytes in flexible monolayers,^31,32^ and characterize binding complexes with nucleic acids.^33^

Previously, we introduced NanoModeler, a webserver that allows its users to prepare the structure and topology files required for atomistic MD simulations of gold NPs (AuNPs) and nanoclusters.^34^ NanoModeler allowed an automatic and standardized protocol for the molecular modeling of AuNPs. However, the generated all-atom representations were limited by the reduced number of experimentally elucidated gold cores. The available crystallographic information restrains the original atomistic approach to 16 AuNPs of under 2.1 nm in diameter, leaving unaddressed the modeling of bigger NPs made of metals other than gold.

In this context, coarse-grained (CG) MD is a computational strategy for simulating large systems by grouping a collection of atoms into individual beads.^35^ Reducing the total number of degrees of freedom in a system thus allows the modeling of longer spatial dimensions with a constant number of interaction sites. According to the force field used, each bead type is assigned a set of parameters to compute the system’s potential energy.^36^ Of the available CG force fields, Martini is one of the most benchmarked and widely used for simulating biomacromolecules and functionalized metal NPs.^37,38^ Some tools currently allow CG simulations of macromolecules like proteins,^39,40^ nucleic acids,^41,42^ and lipids;^43^ however, functionalized NPs must still be prepared through in-house scripting and the efforts of the interested researcher.

Here, we present NanoModeler CG, a new release in the NanoModeler series that allows the building and parametrizing of functionalized metal NPs at a CG resolution. This new version incorporates the principles of CG mapping to address the need for reliable models of metal NPs over 2 nm in size. Indeed, NanoModeler CG supports NPs with cores shaped in eight different geometries (conformed by up to 800,000 beads) coated by monolayers with eight different morphologies. Moreover, this tool produces structure and parameter files of NPs that are compatible with the Gromacs MD engine and the Martini force field by default. In this way, the automatic generation of NP models is brought in line with experimental advances that offer growing synthetic control over metal NPs.

## Results and Discussion

**Figure 1:**
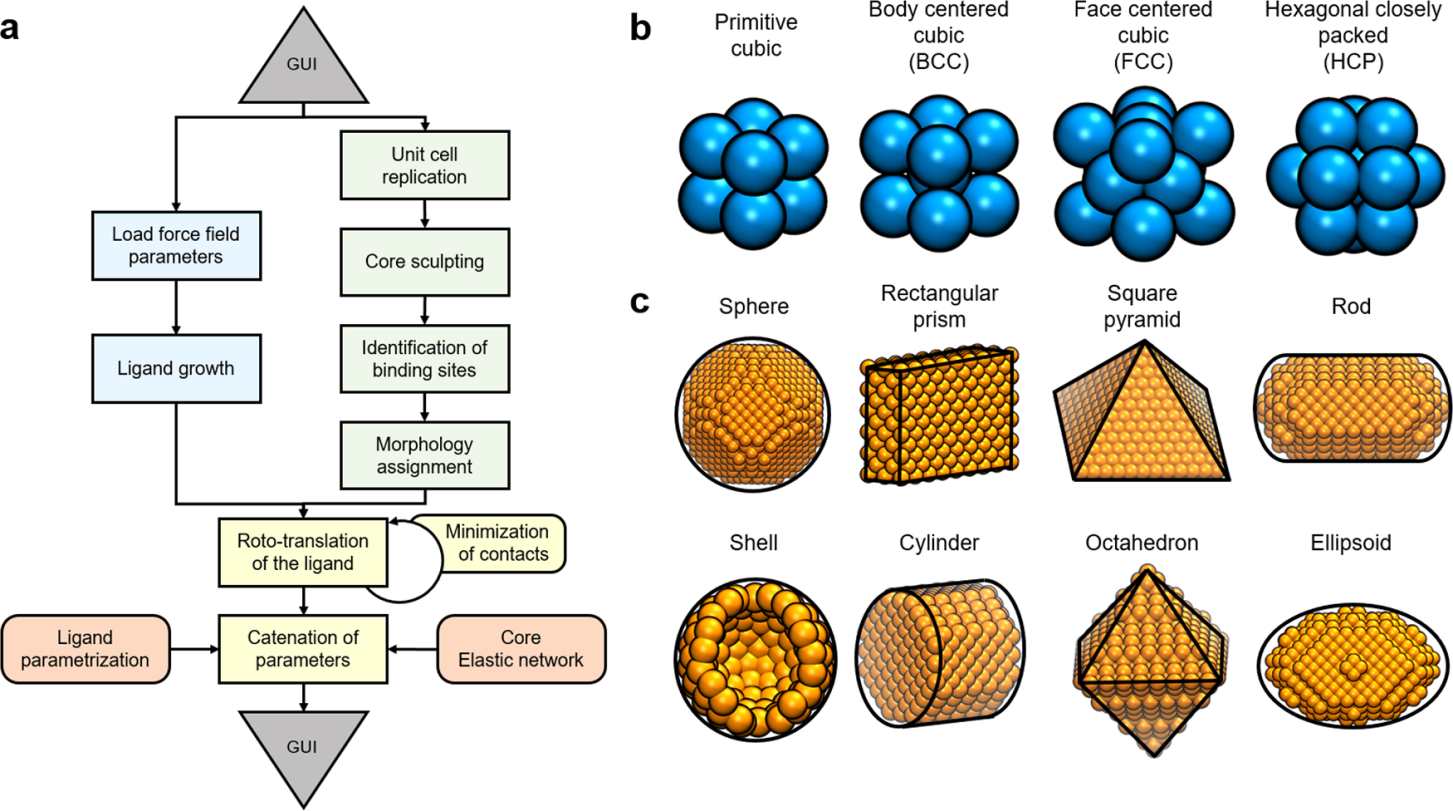
NanoModeler CG’s operations and scope. (a) NanoModeler CG’s general workflow when processing a new job. The graphical user interface (GUI) is in gray, steps involving core beads only are in green, steps involving ligands only are in blue, steps involving core beads and ligands are in yellow, and referrals to force field parameters are in orange. (b) Four unitary cells supported during the core constructions. (c) Eight shapes in which the webserver can sculpt cores.

NanoModeler CG is the second version of the NanoModeler webserver (www.nanomodeler.it).^34^ This version extends the automatic modeling of monolayer-protected metal NPs to CG representations, allowing users to prepare the necessary files for MD simulations. This version also upgrades the previous interface with a new frontend written in HTML, CSS, and JavaScript and a backend written in Python and NodeJS. The webserver offers detailed documentation and tutorials to guide users through the new features (see the Supporting Information). Figure 1a shows NanoModeler CG’s general workflow.

From a structural standpoint, building CG models is linked to a mapping scheme, i.e., the method for encasing multiple atoms into individual interaction sites or beads. Additionally, from a topological perspective, running MD simulations demands a set of bonded and nonbonded parameters assigned to such CG models. In this regard, NanoModeler CG will assign (while not derive) force field parameters according to the user’s input. The webserver is designed to generate highly customizable 3D models of monolayer-protected metal NPs compliant with the Martini force field’s functional form. To achieve this, the structure of the metal NPs is symbolically divided into two components: an inner rigid core and a functionalizing monolayer. The next two sections provide an overview of NanoModeler CG’s customizable features, while a more detailed description of the algorithm can be found in Webserver Building.

### Modeling Nanoparticle Cores

The building of the inner core is key for the modeling of metal NPs because it fixes features that are decisive for many nanotechnological applications such as imaging or drug delivery.^44,45^ Specifically, the core modulates the size, shape, curvature, and mass distribution of the resulting nanoarchitecture.^46,47^ NanoModeler CG can generate cores with up to 800,000 beads. Moreover, because the arrangement of the core beads is material-dependent and unknown *a priori*, the server supports the construction of these cores from the four crystal lattices most encountered in bulk metals (Figure 1b), namely, primitive cubic, body-centered cubic (BCC), face-centered cubic (FCC), and hexagonal closely packed (HCP). The new platform also allows tuning of the core beads’ radius. This allows the use of output models with CG force fields that support any bead mapping strategy.

The webserver models the core as a set of neutral beads that interact with the environment (and each other) through van der Waals forces exclusively. In this way, the hydrophobicity of the core is determined by the bead type selected by the user. Notably, the Lennard-Jones parameters are not explicitly written in the output topology but rather are referred from a secondary file containing the interaction matrix. Although NanoModeler CG offers a copy of Martini’s interaction matrix by default, a single manual modification to the final topology can make the output files easily transferable to a custom force field/interaction matrix. This feature embraces multiple parametrization schemes that other researchers have used to simulate functionalized metal NPs at a CG resolution.^48−50^

The mass of the beads is an additional free parameter that is not explicitly involved in computing the system’s energy but that is needed to calculate the forces exerted on each bead. In CG MD simulations, the mass of the beads must be adjusted according to the chemical moiety that they represent. This is particularly relevant for the core beads because, in general, the default masses in the Martini force field will not add up to the total mass of the core. NanoModeler CG assigns the appropriate mass to each bead by equally distributing the real total mass of the core in the number of beads placed.

The synthesis of shaped metal NPs is now accessible thanks to recent advances in synthetic chemistry. Due to the appearance of anisotropy in their geometry, shaped NPs can display properties that their spherical counterparts cannot. Accordingly, computational methods, in particular MD simulations, are increasingly important ancillary techniques for studying anisotropic metal NPs.^51^ NanoModeler CG can sculpt the crystal lattice into seven different shapes that are experimentally accessible (Figure 1c), namely, sphere, ellipsoid, octahedron, cylinder, rod, rectangular prism, and square pyramid.^52−54^ In addition, the server can build spherical hollow shells. Shells may be preferred over solid spheres because shells display a smoother surface and the absence of inner beads results in a better performance during the computation of nonbonded energetics.^55^ To ensure the core’s rigidity, the server can also impose an elastic network over the core. This elastic network is implemented as a series of bonds between each bead and all its first neighbors in the crystal lattice. The user can also customize the strength of the restraining forces.

### Modeling Nanoparticle Monolayers

The coating ligands are the final component in the parametrization of the NP models. The number of ligands to bind to the core is based on the user-specified grafting density (also known as “ligand footprint”), another experimentally tunable variable over which NanoModeler CG offers full control. In practice, NanoModeler CG grows the ligand molecules from selected superficial core beads. The total number of beads on the core’s surface depends on the size and shape of the NP and on the bead radius parsed. Notably, the ligands are placed around the core, in such a way that the resulting monolayer is as voluminous and sterically unhindered as possible.

Unlike the initial release of our webserver,^34^ NanoModeler CG does not require an input structure file of the coating ligand(s). Instead, the new CG module builds in-site minimized structures of the ligand(s) based on directives (i.e., a parameter modification file) parsed by the user through the graphical user interface (GUI). Therefore, the final model downloaded from the server will lie at a local minimum of bonded energy, making the model more robust and less prone to diverging potential energies during MD simulations (and minimizations). The mass associated with each ligand bead can also be set freely, which guarantees the accurate representation of the atomistic image.

**Figure 2:**
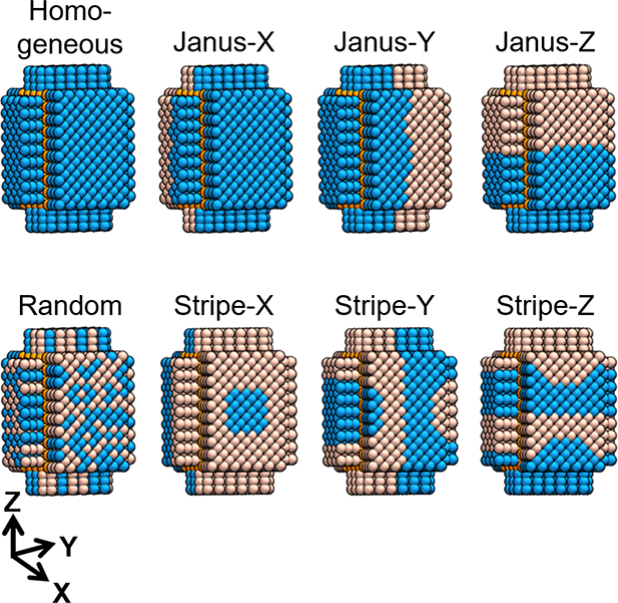
Eight monolayer morphologies supported by NanoModeler CG: homogeneous, random, Janus-X, Janus-Y, Janus-Z, Stripe-X, Stripe-Y, and Stripe-Z. Core beads are in orange, and two example three-bead-long ligands are in blue and pink.

Last, mixed monolayers are gaining attention in the nanotechnology field because they can combine coatings with diverse physicochemical attributes into supramolecular structures with novel properties.^56,57^ To account for this, the coating ligand(s) in NanoModeler CG can be grafted onto the NP cores in seven customizable patterns in addition to the standard single-ligand, homogeneous arrangement. These arrangements are random, Janus-X (along the *X*-axis), Janus-Y, Janus-Z, Stripe-X, Stripe-Y, and Stripe-Z (Figure 2).

### Molecular Dynamics Simulations and Case Study

The topologies generated with NanoModeler CG were validated by reproducing previously published data from experiments and all-atom MD simulations of monolayer-protected gold NPs (AuNPs). Taken together, these test cases illustrate the server’s capabilities and the insights that CG MD simulations of metal NPs can deliver. First, we used 100 ns-long CG MD simulations to compute the monolayer thickness of spherical 10 nm AuNPs coated with different poly(oxyethylene) alkyl ethers (PAE). Second, we calculated the average tilt angle of alkyl chains of different lengths grafted onto spherical 3, 5, and 7 nm AuNPs at various temperatures. Last, we simulated spherical 4.5 nm AuNPs coated with randomly mixed monolayers of polyethylene glycol (PEG) and 11-mercaptoundecanoic acid (MUA).

**Figure 3:**
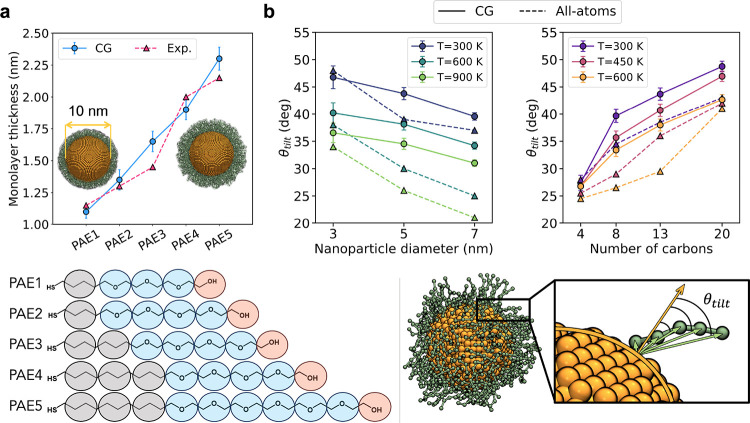
Validation results for models generated with NanoModeler CG. (a) Computed and experimental monolayer widths for 10 nm AuNPs coated with PAE variations of different lengths. The CG mapping scheme is shown in the bottom panel. In our models, the sulfur atoms were considered part of the cores’ anchor beads. The colored circles represent three different bead types and the CG mapping scheme adopted. (b) Tilt angles for alkylthiolated AuNPs derived from CG and all-atom MD simulations. The left plot includes the angles of AuNPs with varying core sizes at 300, 600, and 900 K coated with three-bead (13-carbon) chains. The right plot includes data for 5 nm AuNPs coated with chains of varying lengths and at 300, 450, and 600 K. The bottom panel and its inset illustrate the definition and calculation procedure for the tilt angle θ_tilt_ of a five-bead-long ligand. The angles reported are averaged over all the beads in the ligand, all the ligands in the AuNP, and all the frames in the simulation trajectory. Core beads are in orange, and ligand beads are in green.

For our first test case, PAE-coated AuNPs, we calculated the normalized cumulative radial distribution function (RDF) of the monolayer beads with respect to the core’s center of mass. Then, the monolayer thickness was derived from the limiting radii, within which the monolayer beads are found with a 90% probability (Figure 3a). The computed values are in excellent agreement with the widths measured with differential centrifugal sedimentation (DCS) experiments.^58^ The qualitative trend is perfectly reproduced, with the monolayer becoming wider as the length of the thiols increases. Moreover, the data correspond quantitatively with the experimental values. Specifically, our computed widths display a mean absolute error of 0.11 ± 0.07 nm, which coincides with the expected error from the DCS measurements. These results reveal that the modeling scheme implemented in NanoModeler CG allows the ligands to be properly packed in homogeneous monolayers.

In our second test case, we studied the effect of chain length, core size, and temperature on the chain tilting of alkylthiolated AuNPs. We prepared CG models of 3, 5, and 7 nm AuNPs coated with different alkyl chains, and we compared the chains’ average tilt angle (with respect to a radially outgoing vector) with those estimated from all-atom MD simulations reported elsewhere (Figure 3b, inset).^59^ In their original publication, Ghorai and Glotzer studied AuNPs coated with chains of 4, 8, 13, and 20 carbon atoms. In this work, we model the same aliphatic thiols as one-, two-, three-, and five-bead chains in compliance with Martini’s 4-to-1 mapping scheme. Importantly, in all cases, our CG simulations reproduced the published all-atom trends. For AuNPs coated with 13-carbon chains, the average tilt angle is diminished as the core size increases, which suggests that the models perceive the effect of the core’s curvature on the motion of the ligands (Figure 3b, left). In fact, as the AuNPs’ size increases, the core’s curvature decreases. This reduces the available volume per chain, ushering the ligands into an extended and more organized configuration.

Our simulations on alkylthiolated AuNPs also reproduced a reduction in the average tilt angle as the temperature increases. Here, our data deviate more strongly from the atomistic simulations at elevated temperatures (450 and 600 K, Figure 3b, left).^59^ This effect is likely due to the fewer degrees of freedom in CG models compared to atomistic representations, which leads to fewer microstates and, thus, an underestimation in the conformational entropy of the AuNPs. The dysregulation between enthalpic and entropic contributions in CG force fields has led to the appearance of similar phenomena in systems containing proteins and membranes.^60−62^ Finally, the computed tilt angles also reproduce semiquantitatively the predictions made by the atomistic simulations of 5 nm AuNPs coated with chains of varying length (Figure 3b, right). As before, an increase in temperature (450 and 600 K) results in a decrease in tilt angle. Moreover, if we fix the temperature and focus on the effect of the chain length, it appears that longer alkyl chains more effectively sample their bent configurations (high tilt angles), suggesting an increased flexibility of the terminal section of the ligands.

**Figure 4:**
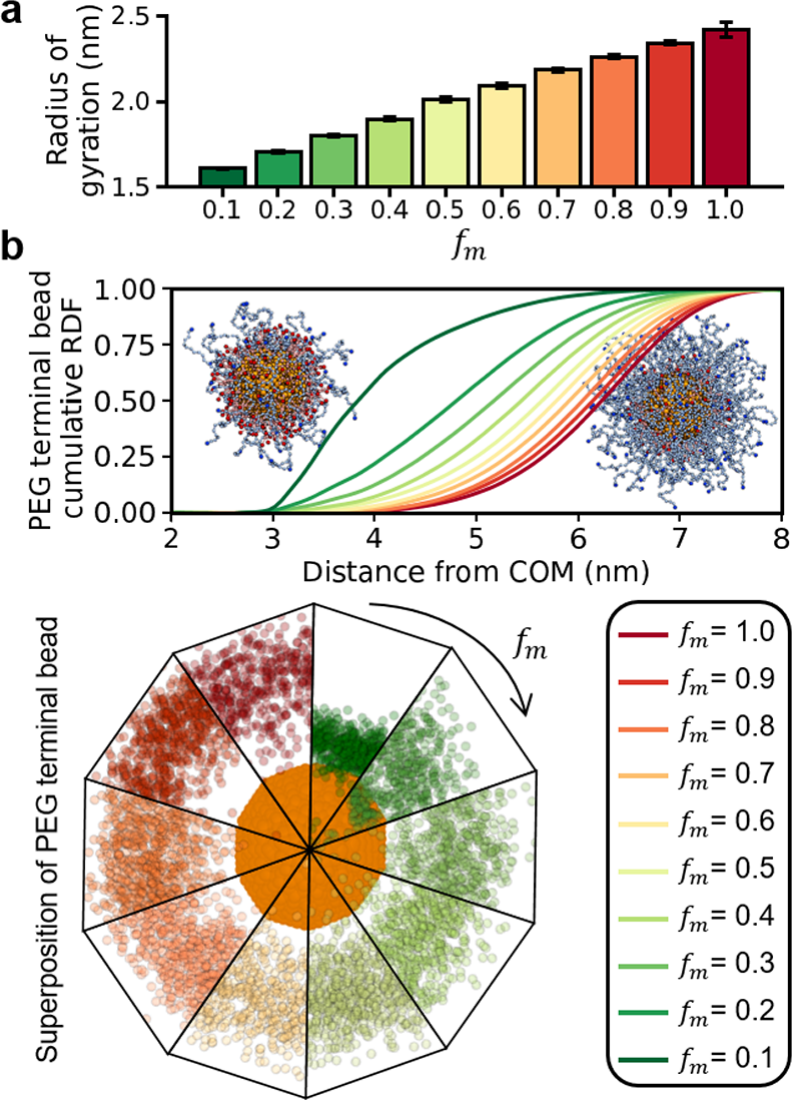
CG MD simulations of 4.5 nm AuNPs coated with a mixed monolayer of PEG and MUA. (a) Radius of gyration of the AuNPs as the fraction of PEG ligands (*f*_m_) increases. (b) Cumulative RDF (normalized to 1.0 at long distances) of the terminal bead in the PEG chains. The panel includes the structure of an AuNP with *f*_m_ = 0.1 (left) and *f*_m_ = 1.0 (right). Core beads are in orange, PEG molecules are in light blue, and MUA molecules are in pink. In the bottom panel, the PEG terminal beads are superimposed for various frames to illustrate the bending of the PEG chains as their relative abundance increases.

As a final test case, we considered AuNPs protected by PEG/MUA monolayers randomly mixed at different fractions (*f*_m_). Dynamic light scattering (DLS) experiments have shown that increasing the abundance of 50 units PEG chains (i.e., higher *f*_m_) leads to a 2-fold increase in the hydrodynamic radius of AuNPs with a 9 nm core.^63^ We thus studied these systems with smaller models that retained the same core-ligand size scale. In detail, we simulated 4.5 nm AuNPs coated by randomly mixed monolayers of 25 units PEG chains and MUA. From our simulations at different values of *f*_m_, we calculated the radius of gyration of the AuNP, a measure that scales linearly with the hydrodynamic radius in globular bodies (Figure 4a).^64^ As demonstrated in DLS experiments, our simulations indicate that higher concentrations of PEG lead to a larger AuNP radius.

We further studied the structural features of PEG/MUA-coated AuNPs by computing the normalized cumulative RDF of the PEG chains’ terminal bead with respect to the core’s center of mass (Figure 4b). As more PEG chains are grafted onto the AuNP, the RDF curves are shifted toward greater distances, indicating a conformational rearrangement of the PEG polymers. As *f*_m_ increases, the PEG chains, which are 21 beads longer than the MUA ligands, form a bulkier monolayer that forces the grafted polymers into an elongated conformation. In contrast, as *f*_m_ decreases, the PEG chains bend inward into a coiled conformation. These contrasting structural features of PEG at different mixing fractions coincide with the brush-to-mushroom transition demonstrated for other PEGylated biological systems.^65,66^

Taken together, the test cases demonstrate the possible applications and studies that one can perform with the NanoModeler CG server. The generated models semiquantitatively reproduce structural features of monolayer-protected metal NPs observed in atomistic simulations and experiments. Moreover, the type of simulation discussed here can also be used to inspect and monitor the conformational rearrangements that lead to variations in experimentally measurable quantities such as the hydrodynamic radius.

## Webserver Building

The NanoModeler webserver comprises two main branches: the frontend and the backend. The frontend is a single-page application developed with Angular-v13, an open-source platform for building desktop and web applications engineered by Google. The user interface embraces Material Design principles through the Angular Material component library, allowing good responsiveness and usage from various devices and platforms. The backend is an aggregation of MicroServices running in Docker containers. Some, like the orchestrator and data persistence layer, are built on top of NodeJS, whereas the code to assemble nanosystems and their topology is written in Python.

### Construction and Parametrization of Packed Cores

The assembly of ligand-coated metal NPs is divided into two processes: the building of the core and the building of the coating molecules. Both processes are customizable by the user. In total, the cores made by NanoModeler CG support four crystal lattices (primitive, body centered cubic, face centered cubic, and hexagonal closely packed), eight shapes (shell, sphere, octahedron, cylinder, rod, pyramid, ellipsoid, and rectangular prism), and eight ligand morphologies (homogeneous, random, Janus-X, Janus-Y, Janus-Z, Stripe-X, Stripe-Y, and Stripe-Z).

**Figure 5:**
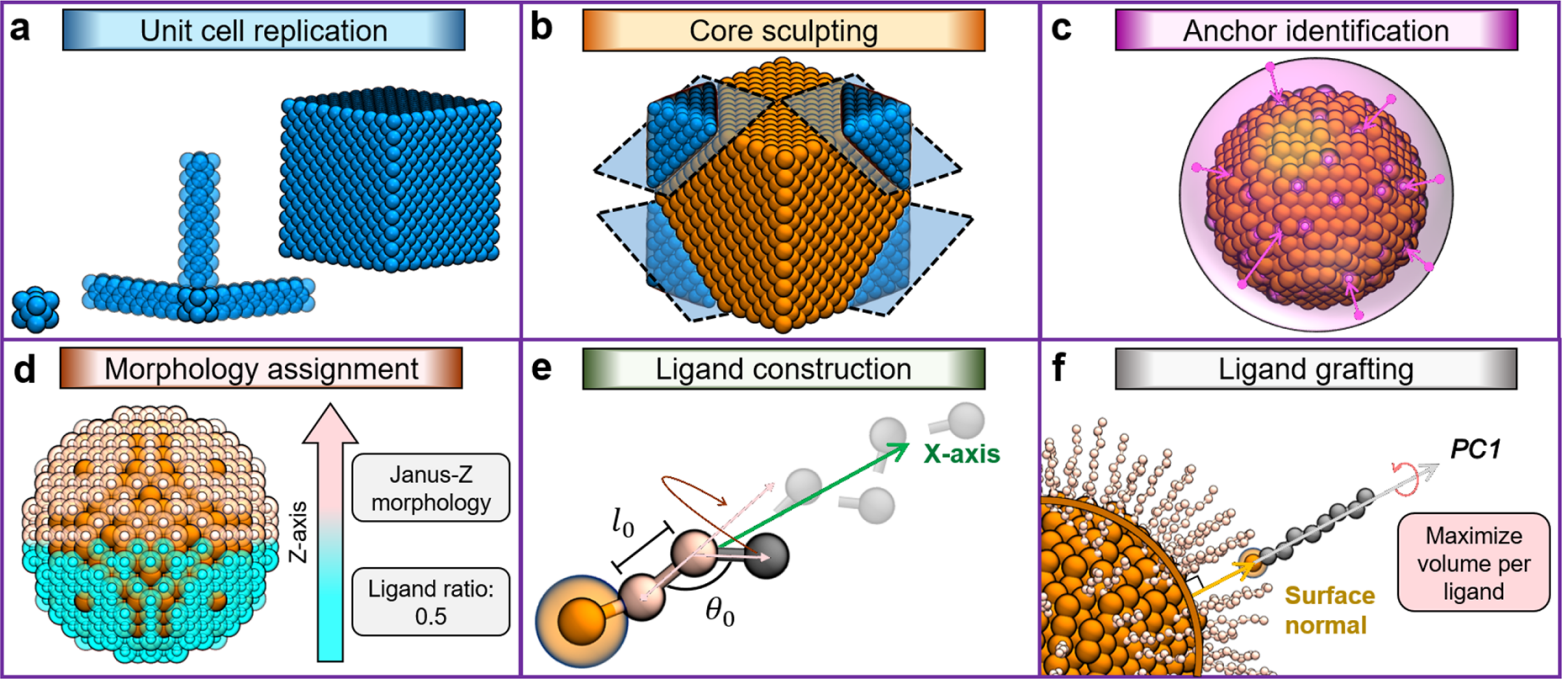
Steps followed by NanoModeler CG when generating the output models. (a) Replication of a unit cell along the *X*, *Y*, and *Z* coordinates. (b) Sculpting of the block into the user-selected shape. (c) Placement of the anchors according to the angular distance between spherically distributed virtual sites and the core’s surface beads. (d) Labeling of the anchors to assign a monolayer morphology. The figure illustrates a Janus-Z morphology with a ligand ratio of 0.5. (e) Construction of the ligand along a reference axis following the equilibrium bond lengths (*l*_0_) and bending angles (θ_0_) provided as inputs. (f) Roto-translation of a ligand aligning its first principal component (PC1) with the vector normal to the core at an anchor’s location. Every ligand is rotated along its molecular axis to maximize the distance to all other beads. Lattice beads are in blue, core beads are in orange, virtual sites are in purple, the anchors of two example ligands are in white and cyan, and the coating ligands are in pink.

The construction of the core follows four steps: (i) the replication of a crystal lattice into a cubic block, (ii) the sculpting of the block into the target shape, (iii) the identification of the anchoring sites for the posterior placing of the ligands, and (iv) the labeling of the anchors. In the first step, a unitary cell is generated in accordance with the crystal motif specified by the user (Figure 5a). The unit cell is replicated in all three dimensions to produce a cubic block that circumscribes the dimensions of the target metal NP. In the special case of a hollow shell, no lattice needs to be specified. In the second step, the block is sculpted into the selected shape (Figure 5b). Note that, depending on the intended shape, the user must provide different geometrical parameters. In detail, a radius must be parsed to build a sphere or shell, an edge length for an octahedron, a radius and length for a cylinder or rod, a base length and height for a pyramid, three semiaxes for an ellipsoid, and three edge lengths for a rectangular prism. In the third step, the pivoting beads for the attachment of the coating ligands are identified (i.e., the anchors, Figure 5c). The total number of anchors, *N*_lig_, is calculated from the grafting density (in nm^2^ per ligand) parsed by the user and the core’s surface area. To select the anchors, *N*_lig_ virtual sites are uniformly placed on a unitary sphere. The distance between points is then maximized following a Metropolis–Hastings algorithm under the constraint that they remain on the sphere. That is, we maximize the sum of distances *D*_u_ subject to the unitary constraints *g*_c, *i*_ (eq 1).
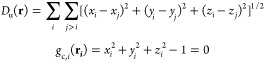
1

The angular distance between these points and the core’s surface beads is then computed. The *N*_lig_ core beads closest to the optimized virtual sites are stored as the anchors. If a bead is selected twice, one of the selections is exchanged for the nearest available surface bead. Here, the vector normal to the core’s surface at each anchor is also stored. In the fourth and final step, the anchors are assigned a flag indicating which ligand to place in each position (Figure 5d). This, in turn, fixes the NP’s ligand morphology. For example, if the morphology is set to “homogeneous”, all anchors will be assigned the same label. In contrast, if a “random” morphology with an occurrence rate of 0.5 is being built, half of the anchors will be assigned the “Ligand 1” flag, and the other half will be recognized as “Ligand 2”. Note that, in the case of a random morphology, the user can ensure reproducible results by setting a random seed. Moreover, the Janus and striped monolayers allow the user to control the ligands’ relative abundance and the number of stripes, respectively.

As with the core, the building of the coating ligands is a three-step sequence: (i) the construction of an individual ligand, (ii) the roto-translation of the ligand around the core, and (iii) the optimization of each ligand’s principal component axis. The algorithm for the first step requires a parameter file. If the user does not provide a parameter file, the ligands’ beads are placed collinearly. In contrast, when the parameters are available, the ligands are still built in an elongated conformation but in accordance with the equilibrium bond lengths and bending angles in the file (Figure 5e). For this, each bead is appended sequentially while simultaneously maximizing the length of the molecule along its main axis (PC1), i.e., minimizing the angle between PC1 and the reference *X*-axis. In the second step, the ligand is roto-translated with a quaternion matrix toward each of the anchoring sites (Figure 5f). This transformation aligns PC1 with the vector normal to the anchor (*vide supra*). Note that this transformation is not unique, and it may result in structural overlaps within the structure. To avoid such internal clashes, in the third step, the ligand is rotated for 20 iterations around PC1, storing the configuration that maximizes the shortest distance between the ligand and the rest of the system.

### 3D Modeling of NPs

The webserver may be used to generate 3D models of monolayer-protected metal NPs (.pdb and .gro files) using the algorithm described above. In addition, the server can also generate topology files for running MD simulations with the Gromacs engine, compliant with the Martini force field’s functional form.^67,68^ For the bonded energy terms, the stretching and bending potentials are modeled by harmonic functions described by a spring constant and an equilibrium (zero) value, whereas torsion potentials are modeled as a sum of periodic functions, each described by an amplitude, an equilibrium value, and a multiplicity. Furthermore, nonbonded interactions include two terms, namely, electrostatics and van der Waals forces. Electrostatics act according to Coulomb’s potential determined by the beads’ partial charge. Van der Waals forces are modeled with a 6–12 Lennard-Jones potential computed according to a transferable and predefined interaction matrix proper of the force field.

The NP bonded parameters are fully customizable by the user by providing a file with force constants, equilibrium values, and multiplicities. Note that the bonded parameters of the ligands cannot be derived exclusively from the bead types. For example, the equilibrium length of a bond cannot be inferred only from the types of the bonded beads. At a coarse-grained resolution, the bonded parameters depend on the chemical nature of the molecules being simulated. Thus, the parameters should be uploaded in an “include topology” (.itp) file following Gromacs’ formatting directives. The server is then able to assign the bonded parameters and bead types to the NP in compliance with the parsed file. This feature makes the final topology compatible with the user’s CG force field of preference. Note that the nonbonded parameters in CG force fields are typically derived from combination rules or fitting schemes unknown *a priori*. For this reason, the final topology file refers to an external database (included in the output) containing the interaction matrix associated with the Martini v2.2refPol force field.^69−71^ If the user wishes to overwrite the parameters of this force field or use a different one, they simply change the line referring to the force field in the final topology (.top) file. In this way, the user has maximum control over the system parameters and the approach remains compatible with the Martini scheme, one of the most used force fields for simulating NPs and biomacromolecules.

To prepare the 3D model, the assignment of parameters is processed sequentially for the internal core first and then the outer monolayer. The CG core must weigh the same as an equivalent atomically detailed representation. For this reason, NanoModeler CG requires the bulk density of the core material as an input. With this information and the volume of the core, the server calculates the real mass of the bulk NP, which is then distributed over the available core beads. NanoModeler CG also allows its users to impose an elastic network over the internal beads to ensure that the shape of the core is maintained throughout a simulation. In doing so, bonds are formed between each of the beads and their nearest neighbors. The number of nearest neighbors varies according to the crystal lattice selected to build the metal NP (i.e., 6 for primitive, 8 for BCC, 12 for FCC, and 12 for HCP). The nearest neighbors are characterized by being located two-bead radii away from the reference site (Figure 1b). Notably, the core beads’ radius and the spring constant of the elastic network are also free parameters for the user to specify. When the core is a hollow shell, the elastic network unites each bead with its six nearest neighbors and the diametrically opposite site (antipodal bead).

The mass and charge of the ligand beads are dictated by the user’s input. NanoModeler CG explicitly writes the mass and charge of all the ligand beads in the resulting NP. The mass and charges of these beads vary according to the mapping scheme chosen by the user and the represented molecular moiety. In contrast, the bonded parameters of the ligands are assigned in a “center out” fashion, starting from the anchoring bead of the core to the tip of the ligands. First, the server identifies the bead types of the bonded pairs, triplets, and quadruplets to assign the bond, angle, and dihedral parameters, respectively. Then, the equilibrium values and spring constants (and multiplicity of dihedrals) are searched in the input parameter file and assigned correspondingly to every copy of the ligand. If the user does not provide parameters for a specific bonded interaction, this is skipped and noted in the job’s final report. Importantly, some CG force fields require multiple energy functions for the same angle or dihedral. This is easily achievable with NanoModeler CG by appending multiple entries for the same bead combination in the proper sections of the input .itp file. For example, if two sets of parameters are parsed for a specific quadruplet of bead types, then two dihedrals (with their respective parameters) will be assigned to these quadruplets.

### MD Simulations

To validate the topologies generated with NanoModeler CG, we aimed to reproduce monolayer widths measured experimentally^58^ and carbon tilt angles computed from all-atom MD simulations.^59^ In addition, we performed simulations for a third set of NPs that shed light on the conformational rearrangements of mixed monolayer AuNPs. First, to reproduce experimental data, we built five AuNPs with a 10 nm spherical core coated by one of the five poly(oxyethylene) alkyl ether (PAE) molecules shown in Figure 3a.^58^ Second, to match data from all-atom MD simulations, we prepared AuNPs with core diameters of 3, 5, and 7 nm coated by three-bead alkyl chains (Figure 3b, left). We also prepared 5 nm cores and coated them with alkyl chains formed by one, two, three, and five beads (Figure 3b, right).^59^ Finally, the mixed monolayer AuNPs consisted of a 4.5 nm core coated by 25-unit-long polyethylene glycol (PEG) chains and 11-mercaptoundecanoic acid (MUA, Figure 4). These AuNPs were built with PEG:MUA ligand ratios (*f*_m_) ranging from 0.1 to 1.0.^63^ All the AuNPs were constructed from FCC lattices with a core bead radius of 0.17 nm. The core beads were modeled as purely hydrophobic moieties using the C1 Martini bead and by applying an elastic network with force constant *k*_b_ = 32,500 kJ mol^–1^ nm^–2^.^29,72^ Moreover, the gold bulk mass density (19.3 g nm^–3^) was passed to the server to calculate the mass of the core’s beads. The coating ligands were grafted at a density of 0.3 nm^2^ per thiol, a typical value for alkylthiolated AuNPs.^46^ The bonded parameters and bead type definitions of all the ligands were taken from Rossi et al.^65,66^

For the MD runs of our first study case (10 nm PAE-coated AuNPs), a simulation box was built to ensure a minimum distance of 2.0 nm between the AuNPs and the box edges. The boxes were then filled with standard Martini water beads.^71^ To relax the solvent around the particles, minimization was carried out using the steepest descent method. The systems were thermalized and pressurized for 5 ns to 300 K and 1 bar in the NPT ensemble using the V-rescale thermostat (τ_B_ = 2.0 ps) and the isotropic Berendsen barostat (τ_P_ = 5.0 ps and κ = 4.5 × 10^–5^ bar^–1^).^73^ Once the systems had reached the intended temperature and pressure, they were simulated for 100 ns coupled to the isotropic Parrinello–Rahman barostat (τ_P_ = 12.0 ps and κ = 4.5 × 10^–5^ bar^–1^).^74^ For the second study case, we simulated 3, 5, and 7 nm alkylthiolated AuNPs in vacuum to match the conditions used by Ghorai and Glotzer in their atomistic simulations.^59^ These systems were initially heated at a constant rate for 0.5 ns using the V-rescale thermostat (τ_B_ = 2.0 ps) in the NVT ensemble at the target temperature (300, 450, or 600 K). The systems were then simulated for 100 ns under the same conditions.

For our final test case, 4.5 nm AuNPs coated with randomly mixed PEG/MUA monolayers, we followed the same workflow as for the PAE-coated AuNPs. Here, however, we used the refPol water model^69,75^ to properly propagate the electrostatic forces of the charged (MUA) ligands. In all our simulations, bonds were constrained using the LINCS algorithm,^76^ an integration time step of 20 fs was used, and frames were saved every 80 ps for posterior analysis. Short-range nonbonded interactions were calculated within a radius of 1.2 nm of each bead. Long-range electrostatic interactions were considered using the fourth-ordered PME method.^77^ All simulations were conducted with Gromacs-v5.1.4.^67,68,78^

Our various simulations allowed us to compare monolayer widths and tilt angles with experimental and all-atom MD simulations, respectively. To calculate the monolayer thickness, we first computed the cumulative normalized radial distribution function of the monolayer beads from the center of mass of the AuNP (eq 2).
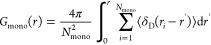
2where *N*_mono_ is the number of beads in the monolayer, *r_i_* is the distance from the *i*th bead to the AuNP’s center of mass, and δ_D_ is the Dirac delta function. Then, we calculated the values of *r*_min_ and *r*_max_ such that *G*_mono_(*r*_min_) = 0.05 and *G*_mono_(*r*_max_) = 0.95, that is, the region where there is a 90% chance of finding the monolayer. The monolayer thickness was computed as *r*_max_ – *r*_min_. The tilt angle of alkylated AuNPs was calculated as the angle between (i) the vector from a ligand’s anchor to a ligand bead and (ii) the vector normal to the core at the ligand’s anchor position (Figure 3b, bottom). The reported values correspond to an average over all the ligand beads, all the ligands, and all the frames. All the error bars were estimated from bootstrap analysis with 1,000 iterations and a sample size of 1% of the original distributions. The trajectory analysis was carried out with a mixture of Gromacs tools and in-house scripts.^79^

## Conclusions

Here, we introduce NanoModeler CG, a webserver for assembling and parametrizing functionalized metal nanoparticles (NPs) at a coarse-grained (CG) resolution. NanoModeler CG automates and standardizes the modeling of metal NPs larger than 2 nm. NanoModeler CG extends the original (atomistic) methodology to metal NPs with nonspherical cores of up to 800,000 beads coated by a broader range of monolayer morphologies, consistent with growing synthetic control. Similar to the first release for atomistic NP models, NanoModeler CG stratifies the building of the 3D models and the parameter assignment by treating the inner metallic core followed by the coating monolayer. The construction of the inner metallic core supports four different crystal unit cells that can be combined with eight different core shapes. The coating ligands are built in site, and a model that lies at a local minimum of bonded potential energy is built with bonded parameters parsed by the user.

To illustrate some of the insights that NanoModeler CG and the CG methodology can provide, we modeled three representative NP test systems, sampled in several different flavors. MD simulations of 10 nm PAE-coated AuNPs reproduced the experimental monolayer thickness with a precision of 0.1 nm. Similarly, simulations of alkylthiolated AuNPs reproduced semiquantitatively the tilt angles of the coating thiols at different core sizes, thiol chain lengths, and temperatures. Finally, our simulations of 4.5 nm mixed monolayer AuNPs rationalized the decrease in the hydrodynamic radius of PEG/MUA-coated AuNPs in terms of a brush-to-mushroom transition of the passivating PEG chains. Taken together, these results demonstrate that NanoModeler CG is an effective tool for studying structural and dynamical features of functionalized metal NPs larger than 2 nm. Ultimately, NanoModeler CG facilitates access to the computational modeling of (large) monolayer-protected metal NPs, thus aiding their knowledge-based design.

## Supporting Information Available

List of tutorials on how to use NanoModeler CG (PDF)
