# Supplementary material for: NanoModeler CG: A Tool for Modeling and Engineering Functional Nanoparticles at a Coarse-Grained Resolution
Source: J Chem Theory Comput. 2023 Feb 16;19(5):1582–91. doi: 10.1021/acs.jctc.2c01029 (PMC10018737; doi:10.1021/acs.jctc.2c01029)
Supplement: Supplementary file 1 — ct2c01029_si_001.pdf [file ct2c01029_si_001.pdf]

Supporting Information

NanoModeler CG: A tool for modeling and engineering functional nanoparticles at a coarse-grained resolution

Sebastian Franco-Ulloa[1, 2], Laura Riccardi[1], Federico Rimembrana[1], Edwin Grottin[1], Mattia Pini[1], and Marco De Vivo[*,1]

1. Molecular Modeling and Drug Discovery Lab, Istituto Italiano di Tecnologia, via Morego 30, 16163 Genova, Italy.
2. Expert Analytics, Møllergata 8, 0179 Oslo, Norway.

Corresponding author:
Dr. Marco De Vivo – Email: marco.devivo@iit.it

Overview

NanoModeler-CG is a web server that allows you to build coarse-grained (CG) monolayer protected nanoparticles.

With NanoModeler you can obtain a coordinate (.gro file formats) and parameter files (.top file format). The structure and topology files offered here are ready for use with the Gromacs MD engine.

The parameters offered here are based on the MARTINI force field.

NanoModeler-CG does not require an input structure file of the coating ligand(s). Instead, the CG module builds on-site minimized structures of the ligand(s) based on directives parsed by the user through the graphical user interface (GUI).

NanoModeler-CG currently supports NPs with cores shaped in eight different geometries (up to 800 000 beads) coated by monolayers with eight different morphologies.

Job Options

When running a job in NanoModeler-CG, you can modify several options to customize the output. Here, we explain what each field means. All options must be specified from the GUI. This improves the user experience and reduces the number of files that the user has to provide.

Below, you will find the names, data types, and important information on all the current options:

- Job Name (string): The name given to the job in the queueing system. Each job must have a unique name. This field does not determine the name of the output files.
- Description (string): Description of the running job. With this field, the user can more easily track each job.
- Core Shape (string): One core must be selected from the drop-down list in the field and the corresponding parameters should be inserted:
    - Cylinder: “Radius” and “Length” of the cylinder (nm). Use “.” for decimals.
    - Ellipsoid: “Semiaxis (X)”, “Semiaxis (Y)”, and “Semiaxis (Z)” of the ellipsoid (nm). Use “.” for decimals.
    - Octahedron: “Edge-length” of the octahedron (nm). Use “.” for decimals.
    - Pyramid: “Base Edge-length” and “Height” of the square pyramid (nm). Use “.” For decimals.
    - Rectangular Prism: “Length (X)”, “Length (Y)”, and “Length (Z)” of the rectangular prism (nm). Use “.” For decimals.

    - Rod: “Capping Sphere Radius” and “Cylindrical Length” of the midsection (nm). Use “.” For decimals.
    - Shell: “Radius” of the hollow shell (nm). Use “.” For decimals.
    - Sphere: “Radius” of the sphere (nm). Use “.” For decimals.
- Crystal motif (string): Crystal motif used for building the core. One core must be selected from the drop-down list in the field. NanoModeler-CG supports the four crystal lattices most encountered in bulk metals: “BCC”, “FCC”, “HCP”, and “Primitive”. With “BCC”, a body-centered cubic cell is used. With “FCC”, face-centered cubic cell is used. With “HCP”, hexagonal close packed cell is used. With “Primitive”, a single unit cell is used.
- Bulk Density (float): Mass density of the core's bulk material (g cm^{-3}). Use “.” for decimals.
- Graft Density (float): Average surface area per ligand (nm^2 per ligand). Use “.” for decimals.
- Bead Type (string): Alphanumeric bead type to assign to the core beads. Maximum 5 characters.
- Bead Radius (float): Radius of the core beads (nm). Use “.” for decimals. The Bead Radius value should be smaller than the values inserted for the Core Shape, above.
- Elastic Network (boolean): (De)activate a harmonic elastic network connecting each core bead with its nearest neighbors. When “Elastic Network“ is activated, insert “Force Constant“ value for the bonds in the elastic network (kJ mol^{-1} nm^{-2}). Use “.” for decimals.

Ligand 1 and Ligand 2 boxes:

- Bead Type (string): Sequence of (alphanumeric) bead types conforming the selected ligand from the inside out. Maximum 5 characters.
- Charge (float): Signed charge for each bead type in the selected ligand from the inside out (*e*). Use “.” for decimals.
- Mass (float): Mass for each bead type in the selected ligand from the inside out (a.m.u.). Use “.” for decimals.
- N-count (integer): Number of repetitions of each bead type to build the selected ligand from the inside out.

Select "Abort" to cancel the newly inserted values, "Clear" to restore the previously saved values, "Submit" to save the current values. If you need, you can edit the values before submitting the job to the server.

Mixed-Monolayer box:

This box appears when parameters for Ligand 1 and Ligand 2 are provided.

- Morphology (string): If you wish to build a mixed-monolayer protected nanoparticle (i.e., two ligand structure files are supplied), you can use this option to select the ligands arrangement. NanoModeler supports three arrangements: “Random”, “Janus”, and “Stripe”. With “Random”, Ligand 1 and Ligand 2 are randomly distributed on the core. With “Janus”, the nanoparticle is partitioned in two hemispheres (along X, Y or Z axis), each coated with a different ligand. With “Stripe”, the beads in the core are sorted into intercalating domains along X, Y or Z axis.
- Ligand 1 Fraction (float): This field is only enabled if “Morphology” is set to “Random” or “Janus”. Enter a value between 0 and 1 to indicate what fraction of Ligand 1 to use. If the value is set to 1 or 0, a homogeneous monolayer is obtained for Ligand 1 or Ligand 2, respectively.
- Random Seed (integer): This field is only enabled when “Morphology” is set to “Random”. Specify a random seed to generate the distribution of Ligand 1 and Ligand 2. Different seeds result in different output structures. If no value is given, it will be selected randomly. The selected seed is always prompted to the user once the job has finished.
- Stripes (integer): This field is only enabled when “Morphology” is set to “Stripe”. Specify the number of stripes in which to allocate Ligand 1 and Ligand 2.

Additional Options box:

- Force-Field Modification File (file): A parameters file (.itp file format) for overwriting or completing the default parameters containing the bonded parameters of the whole system (i.e., core and ligands). For more information on the .itp file format please refer to the Gromacs User Guide. If an .itp file is not provided, Nanomodeler-CG will create only the .gro file of the coated nanoparticle. If an .itp file is provided, Nanomodeler-CG will create the .gro file and the .top file of the coated nanoparticle. You will find a folder called "MARTINI". The .itp files in this directory were taken from: the Martini's website, here. We thank the authors for allowing us to make these files downloadable directly from NanoModeler. When you use these files, please cite the references listed in the header of each file.

Test cases - Tutorial

To use NanoModeler-CG, you will need to create an account. Once you are logged in, you can access the job submission page by clicking on the “Let’s submit your first job!” button on the homepage, or the “Submit your first job!” button that is always visible in the upper-right corner.

Below, you will find three tutorials with different input files. You can follow the tutorials and generate your own files side-by-side, or you can download all the input and output files here. Let’s get started!

Test case 1: Ligands with a single bead (homogeneous monolayer)

In this first scenario, we will generate a Rod gold nanoparticle coated by a short alkyl chain (ALK).

Several parameters need to be provided before submitting the job:

1. Name and description: Once you are on the job submission page, begin by giving your job a name (mandatory) and description (optional).
2. Core: To build the core, several parameters are required. 8 geometrical shapes are available and each one has different parameters associated with it. From the "Core Shape" drop down menu, select "Rod" and set the "Capping Sphere Radius" to 1 nm and the "Cylindrical Length" to 3 nm. For this tutorial, we consider a 1-to-1 mapping of the gold core. Select the FCC lattice organization of gold from the drop down "Crystal Motif" menu and set the "Bulk Density" to 19.3 g cm^{-3}. The core beads are modeled as purely hydrophobic moieties using the C5 Martini bead with a "Bead Radius" of 0.166 nm. Activate the "Elastic Network" option and set the "Force Constant" to 32 500 kJ mol^{-1} nm^{-2}. The "Graft Density" defines the density of the coating ligands on the core's surface. In this example, we set it to 0.3 nm^2 per ligand.
3. Ligand 1: The Ligand 1 box contains all the information about the first coating ligand type. To build the ALK ligand we will use the bead builder once, to map the $(CH_2)_3CH_3$ ligand to one bead. Select "Add a bead" and activate the "Bead Builder" box. Set the first Bead Type as C1, the Charge as 0, the Mass as 57, and the number of repetitions as 1. To accept the parameters click "Submit", while if you wish to return all fields to their default values click the "Reset" button. The parameters of the bead can be modified by clicking the "Edit" button.
4. Ligand 2: In this tutorial, we are generating a homogeneous monolayer-protected nanoparticle, so we will not build Ligand 2.
5. Mixed monolayer: In this tutorial, we only built Ligand 1, so the Mixed Monolayer box is disabled by default.
6. Force field parameters: Upload the .itp file that you can find here. Remember: if no .itp file is uploaded, you will obtain only the .gro file of the system and not the .top file.
7. Submission: To submit your job, click the "Submit" button at the bottom of the page.
8. After the job is submitted: Once the job is submitted you will be redirected to your profile. There, you will find a list of your jobs and their current status. You may wait for the job to finish, but you will also receive an email to your registered address, informing you when it has finished.
9. Results download and availability: To download the results, log on to your NanoModeler account and access your job list. Alternatively, click on the link in the notification email. Both options will take you to a message with information about the run and, if the job failed, additional information for troubleshooting. This message also contains all the input

options used to execute the job. Your results are available for download as a zipped file for up to 10 days after job submission. If you would like to take a look at the results from this tutorial, feel free to download an example output from here.

And that's it. You're all set to run your MD simulations. Good luck!

Test case 2: Ligands with multiple different beads (homogeneous monolayer)

In this second scenario, we will generate a spherical gold nanoparticle of 10 nm coated by molecules of polyoxyethylene alkylethers (PAE).

Several parameters need to be provided before submitting the job:

1. Name and description: Once you are on the job submission page, begin by giving your job a name (mandatory) and description (optional).
2. Core: To build the core, several parameters are required. 8 geometrical shapes are available and each one has different parameters associated with it. From the "Core Shape" drop down menu, select "Sphere" and set the "Radius" to 5 nm. For this tutorial, we consider a 1-to-1 mapping of the gold core. Select the FCC lattice organization of gold from the drop down "Crystal Motif" menu and set the "Bulk Density" to 19.3 g cm^{-3}. The core beads are modeled as purely hydrophobic moieties using the C5 Martini bead with a "Bead Radius" of 0.166 nm. Activate the "Elastic Network" option and set the "Force Constant" to 32 500 kJ mol^{-1} nm^{-2}. The "Graft Density" defines the density of the coating ligands on the core's surface. In this example, we set it to 0.5 nm^2 per ligand.
3. Ligand 1: The Ligand 1 box contains all the information about the first coating ligand type. To build the PAE ligand we will use the bead builder three times: the first time for the inner $(CH_2)_3$ beads, the second time for the CH_2OCH_2 units, and the third time for the CH_2OH capping. For each bead type, starting from the closest one to the core, select "Add a bead" and activate the "Bead Builder" box. Set the first Bead Type as C1, the Charge as 0, the Mass as 56, and the number of repetitions as 1. Set the second Bead Type as EO, the Charge as 0, the Mass as 44, and the number of repetitions as 3. Set the third Bead Type as SP2, the Charge as 0, the Mass as 31, and the number of repetitions as 1. To accept the parameters click "Submit", while if you wish to return all fields to their default values click the "Reset" button. The parmeters of each bead can be modified by clicking the "Edit" button.
4. Ligand 2: In this tutorial, we are generating a homogeneous monolayer-protected nanoparticle, so we will not build Ligand 2.
5. Mixed monolayer: In this tutorial, we only built Ligand 1, so the Mixed Monolayer box is disabled by default.

6. Force field parameters: Upload the .itp file that you can find here. Remember: if no .itp file is uploaded, you will obtain only the .gro file of the system and not the .top file.
7. Submission: To submit your job, click the "Submit" button at the bottom of the page.
8. After the job is submitted: Once the job is submitted you will be redirected to your profile. There, you will find a list of your jobs and their current status. You may wait for the job to finish, but you will also receive an email to your registered address, informing you when it has finished.
9. Results download and availability: To download the results, log on to your NanoModeler account and access your job list. Alternatively, click on the link in the notification email. Both options will take you to a message with information about the run and, if the job failed, additional information for troubleshooting. This message also contains all the input options used to execute the job. Your results are available for download as a zipped file for up to 10 days after job submission. If you would like to take a look at the results from this tutorial, feel free to download an example output from here.

And that's it. You're all set to run your MD simulations. Good luck!

Test case 3: Two different ligands (mixed monolayer)

In this third tutorial, we will generate a spherical gold nanoparticle of 10 nm coated by randomly mixed monolayer of polyethylene glycol (PEG) and 11-mercaptoundecanoic acid (MUA) molecules.

Several parameters need to be provided before submitting the job:

1. Name and description: Once you are on the job submission page, begin by giving your job a name (mandatory) and description (optional).
2. Core: To build the core, several parameters are required. 8 geometrical shapes are available and each one has different parameters associated with it. From the "Core Shape" drop down menu, select "Octahedron" and set the "Edge-length" to 3 nm. For this tutorial, we consider a 1-to-1 mapping of the gold core. Select the FCC lattice organization of gold from the drop down "Crystal Motif" menu and set the "Bulk Density" to 19.3 g cm^{-3}. The core beads are modeled as purely hydrophobic moieties using the C5 Martini bead with a "Bead Radius" of 0.166 nm. Activate the "Elastic Network" option and set the "Force Constant" to 32 500 kJ mol^{-1} nm^{-2}. The "Graft Density" defines the density of the coating ligands on the core's surface. In this example, we set it to 0.5 nm^2 per ligand.
3. Ligand 1: The Ligand 1 box contains all the information about the first coating ligand type. To build the PEG ligand we will use the bead builder twice: the first time for the inner

beads and the second time for the terminal one. For each bead type, starting from the closest one to the core, select “Add a bead” and activate the “Bead Builder” box. Set the first Bead Type as C1, the Charge as 0, the Mass as 52, and the number of repetitions as 3.

4. The second time, we will add the terminal bead: set the Bead Type as Qa, the Charge as -1, the Mass as 80, and the number of repetitions as 1. To accept the parameters click “Submit”, while if you wish to return all fields to their default values click the “Reset” button. The parameters of each bead can be modified by clicking the “Edit” button.
5. Ligand 2: The Ligand 2 box contains all the information about the second coating ligand type. To build the MUA ligand we will use the bead builder twice: the first time for the inner beads and the second time for the terminal one. For each bead type, starting from the closest one to the core, select “Add a bead” and activate the “Bead Builder” box. Set the first Bead Type as EO, the Charge as 0, the Mass as 44, and the number of repetitions as 24. The second time, we will add the terminal bead: set the Bead Type as SP2, the Charge as 0, the Mass as 31, and the number of repetitions as 1. To accept the parameters click “Submit”, while if you wish to return all fields to their default values click the “Reset” button. The parameters of each bead can be modified by clicking the “Edit” button.
6. Mixed monolayer: As we inserted the parameters of Ligand 1 and Ligand 2, the "Mixed Monolayer" box is activated. Select a "Random" distribution from the drop-down menu and set the "Ligand 1 Fraction" to 0.7 to have a majority of Ligand 1 on the nanoparticle. We leave the "Random Seed" field empty, which will make NanoModeler select it randomly.
7. Force field parameters: Upload the .itp file that you can find here. Remember: if no .itp file is uploaded, you will obtain only the .gro file of the system and not the .top file.
8. Submission: To submit your job, click the “Submit” button at the bottom of the page.
9. After the job is submitted: Once the job is submitted you will be redirected to your profile. There, you will find a list of your jobs and their current status. You may wait for the job to finish, but you will also receive an email to your registered address, informing you when it has finished.
10. Results download and availability: To download the results, log on to your NanoModeler account and access your job list. Alternatively, click on the link in the notification email. Both options will take you to a message with information about the run and, if the job failed, additional information for troubleshooting. This message also contains all the input options used to execute the job. Your results are available for download as a zipped file for up to 10 days after job submission. If you would like to take a look at the results from this tutorial, feel free to download an example output from here.

And that’s it. You’re all set to run your MD simulations. Good luck!
